# The *PHR* Family: The Role of Extracellular Transglycosylases in Shaping *Candida albicans* Cells

**DOI:** 10.3390/jof3040059

**Published:** 2017-10-29

**Authors:** Laura Popolo, Genny Degani, Carlo Camilloni, William A. Fonzi

**Affiliations:** 1 Department of Biosciences, University of Milan, Via Celoria 26, Milan 20133, Italy; genny.degani@unimi.it (G.D.); carlo.camilloni@unimi.it (C.C.); 2 Department of Microbiology & Immunology, Georgetown University, Washington, DC 20057, USA; fonziw@georgetown.edu

**Keywords:** cell wall assembly, β-(1,3)-glucanosyltransferases, morphogenesis, cell wall stress

## Abstract

*Candida albicans* is an opportunistic microorganism that can become a pathogen causing mild superficial mycosis or more severe invasive infections that can be life-threatening for debilitated patients. In the etiology of invasive infections, key factors are the adaptability of *C. albicans* to the different niches of the human body and the transition from a yeast form to hypha. Hyphal morphology confers high adhesiveness to the host cells, as well as the ability to penetrate into organs. The cell wall plays a crucial role in the morphological changes *C. albicans* undergoes in response to specific environmental cues. Among the different categories of enzymes involved in the formation of the fungal cell wall, the GH72 family of transglycosylases plays an important assembly role. These enzymes cut and religate β-(1,3)-glucan, the major determinant of cell shape. In *C. albicans,* the *PHR* family encodes GH72 enzymes, some of which work in specific environmental conditions. In this review, we will summarize the work from the initial discovery of *PHR* genes to the study of the pH-dependent expression of *PHR1* and *PHR2*, from the characterization of the gene products to the recent findings concerning the stress response generated by the lack of GH72 activity in *C. albicans* hyphae.

## 1. Introduction

*Candida albicans* is routinely found as a benign commensal inhabitant of human skin and various mucosal surfaces of the alimentary and genitourinary tract [1]. However, as an opportunistic fungal pathogen it is a frequent agent of non-invasive infections and life-threatening invasive infections in immune-compromised patients [2]. It has been estimated that *C. albicans* causes >400,000 life-threatening infections per year worldwide, with mortality rates of 46–75% [3]. Despite being restricted to the human host as an ecological niche, *C. albicans* is nonetheless exposed to diverse and dynamic microenvironments (mucosae, skin, gastrointestinal tract), which it must sense and to which it must constantly adapt. The interface of this constant exchange is the cell wall. The fungal cell wall is a dynamic structure that changes in response to environmental stresses, including host–pathogen interaction [4,5]. The most overt cell wall changes are manifested in the morphological transitions of *C. albicans* between yeast, pseudohyphae and hyphae, transitions that are critical to its pathogenicity [6].

From a practical perspective, the fungal cell wall has long been considered a desirable target of antifungal drugs given its essentiality and unique structure and biosynthesis relative to the host. The value of this target was demonstrated with the introduction of the echinocandins, which inhibit glucan synthase, the enzyme responsible for production of the major cell wall polysaccharide β-(1,3)-glucan [7]. This glucan is largely responsible for the integrity and shape of the cell wall. The steps leading from the initial synthesis of β-(1,3)-glucan to its modification, interconnection, and incorporation into the three-dimensional architecture of the cell wall remain poorly defined [8]. However, the GH72 transglycosylases are important players in this complex assembly process. The absence of these enzymes has severe consequences, altering growth, morphology, and, virulence. In *C. albicans* the GH72 enzymes are encoded by the *PHR* gene family, some of which are expressed in response to specific environmental stresses. In this review, we will summarize the work from the initial discovery of *PHR* genes to the study of the pH-dependent expression of *PHR1* and *PHR2*, from the characterization of the gene products to the recent findings concerning the stress response generated by the lack of GH72 activity in *C. albicans* hyphae. Elucidating these processes will lead to a fuller understanding of cell wall dynamics and may suggest new therapeutic approaches to this problematic pathogen.

## 2. Identification of the *PHR* Multigene Family

Discovery of the initial members of the *PHR* gene family in *C. albicans*, like the discovery of the orthologous *GAS* genes in *Saccharomyces cerevisiae*, was a fortuitous outcome of studies unrelated to fungal cell wall biosynthesis or structure. Gas1p was first identified as a cell-cycle-regulated protein [9], subsequently shown to be glycosylated [10] and one of the predominant glycosylphosphatidyl inositol (GPI)-linked proteins of the yeast plasma membrane [11,12]. In contrast, *PHR1* was uncovered in a study designed to identify genes expressed specifically in the yeast or hyphal growth phase [13]. The morphology of *C. albicans* can be controlled in vitro by altering culture conditions, pH and temperature being two important variables. In a screening for differentially expressed genes, cells were maintained in the yeast morphology by culturing at 25 °C, pH 4.5 and induced to form germ tubes, the initial phase of hyphal outgrowth, at 37 °C, pH 6.5 [13]. Differential hybridization of cDNA prepared from these cultures identified morphology-specific genes as well as one gene regulated specifically in response to the pH of the culture medium [14]. This gene was designated *PHR1* (pH responsive). DNA sequence analysis showed *PHR1* to be closely related to *GAS1* [14] and heterologous complementation of a *GAS1* null mutant demonstrated their functional equivalence [15].

The existence in *C. albicans* of additional *PHR1*-related genes was suggested by the presence of multiple proteins cross-reactive with anti-Phr1p antisera and the pH-conditional phenotype of *phr1* null mutants [14]. One of the presumptive paralogs was identified by PCR using degenerate primers that targeted sequences conserved between *PHR1* and *GAS1* to amplify related sequences from template DNA of a *phr1* deletion mutant [16]. A single PCR product was obtained. Isolation and sequencing of the corresponding gene showed it to encode a protein co-linear with Phr1p and about 53% identical. This gene was designated *PHR2*. Although multiple primer sets were tested, *PHR2* was the only gene identified.

The third member of the *PHR* family, *PHR3*, was found by chance sequencing of an EST library clone [17], while two additional family members were identified by in silico means. The latter two, *PGA4* and *PGA5*, were found by an algorithmic search of the *C. albicans* genome designed to recognize putative GPI-modified proteins [18], thus their names, predicted GPI-anchored proteins [18]. These three genes are more divergent than *PHR1* and *PHR2*, the encoded proteins are only 25–35% identical to Phr1p and Phr2p and to each other [17]. *PHR3* is distinguished as the only gene in the family to contain an intron and to encode a protein lacking an identifiable GPI attachment site.

### Expression Pattern of PHR Multigene Family

Structural divergence within the PHR family is accompanied by an even greater divergence in expression pattern. As noted above, *PHR1* expression varies as a function of culture pH, independent of culture medium composition, temperature, and cell morphology [14]. Expression, as measured by mRNA levels, is greatest in culture medium adjusted to an alkaline pH of 7.5–8.0 and shows a graded decline in parallel with culture pH to undetectable levels at pH 5.0 [14]. Remarkably, expression of *PHR2* not only responds to ambient pH, but the response is the precise inverse of *PHR1* [16]. Expression is highest at pH 4.0 and is reduced as ambient pH is increased [16]. The pH-conditional expression of these genes is regulated by a zinc-finger containing transcription factor Rim101p, which is proteolytically activated at ambient alkaline pH and induces transcription of *PHR1* and represses expression of *PHR2* [19,20,21,22,23].

In contrast to *PHR1* and *PHR2*, expression of the other family members is completely independent of ambient pH. *PHR3* and *PGA5* are constitutive and weakly expressed [17]. Transcript levels of *PGA4* are significantly higher and, although pH has no influence, the presence of serum causes a transitory 3- to 4-fold induction [17]. Expression is also increased several fold in a reconstituted human epithelial model and in vivo [17]. These transcriptional differences are directly reflected at the protein level. Phr1p is greatly enhanced in the walls of cells cultured at pH 7, whereas Phr2p is absent, and, conversely, Phr2p is found when cultures are grown at pH 4 [24]. Pga4p is present under both conditions [24].

## 3. Molecular Features and Localization of Phr Proteins

### 3.1. Phr Proteins Are *C. albicans* Representatives of Family GH72

The deduced amino acid sequences of the *PHR* genes initially provided no clue to function except for a distant relationship with bacterial β-glycosidases [25]. The proteins encoded by *PHR1* and *PHR2* showed a high degree of amino acid identity (>50%) with Gas1p of *S. cerevisiae*, the Gel1 protein of the filamentous fungus *Aspergillus fumigatus* (*Af*Gel1) and Epd1p of *C. maltosa* (*Cm*Epd1). Gel/Phr/Gas/Epd proteins became the founders of a new group of glycoside hydrolases (GH) classified as family 72 in the database of carbohydrate-active enzymes (CAZy) and whose activity will be described in Section 3.1.1. Family GH72 grew rapidly thanks to the progress of several sequencing projects of yeast and fungal genomes, and currently counts hundreds of members distributed in many species, from *Neurospora crassa* to *Aspergillus* sps., from *Candida* sps. to plant symbiotic fungi such as *Tuber melanosporum* or phytopathogens such as *Magnaporthe oryzae* [26]. These enzymes are unique to the Kingdom Fungi. For simplicity throughout this review, GH72 proteins will be indicated by a prefix of the genus and species of origin except Phr of *C. albicans* and Gas of *S. cerevisiae*.

Interestingly, GH72 enzymes are characterized (i) by a high degree of redundancy within the same species and (ii) by the existence of orthologs across all species so far examined [27]. The most striking examples of redundancy are: the Gel family of *A. fumigatus* (*Af*Gel1p to *Af*Gel7p), the Gas family of *S. cerevisiae* (Gas1p to Gas5p) and the Gel family of *N. crassa* (*Nc*Gel1 to *Nc*Gel5). A notable exception is the fungal pathogen *Cryptococcus neoformans*, and in general Basidiomycota, which have only one GH72-encoding gene [27].

The expansion of gene families encoding GH72 enzymes likely reflects a genetic adaptation to different environmental conditions and ecosystems, in addition to functional specialization of duplicated genes. Cell reshaping occurs during the complex morphological transitions a fungus typically undergoes in its life cycle (yeast/hyphal growth, spore or conidiophore formation) and requires the expression of different paralogs. Well-known examples of a dynamic interplay among paralogs during the fungal life cycle were described for the *GAS* genes of *S. cerevisiae*, the *gas+* genes of *Schizosaccharomyces pombe*, and the *GEL*s of *Neurospora crassa* and of *A. fumigatus* [28,29,30,31,32]. Gene redundancy occurs not only in microorganisms that underwent whole genome duplication, e.g., *S. cerevisiae*, but other species as well. The observed co-expression of two or more paralogs during the same stage of the life cycle guarantees a back-up system able to compensate for the loss of one of the paralogs. Taking into consideration the evolutionary cost of keeping duplicated genes in a genome, these observations accentuate the importance of GH72 activity for fungal morphogenesis.

#### 3.1.1. Catalytic Activity of the GH72 Enzymes: *Af*Gel1, Gas1, and Phr1-Phr2 Proteins

In 2000, the Laboratoire des *Aspergillus* led by Jean Paul Latgé, using laminarin-derived laminarioligosaccharides (β-(1,3)-linked glucose residues) of different length as substrate and HPAEC analysis of the reaction products, demonstrated for the first time that GH72 proteins were catalytically active in processing β-(1,3)-glucan molecules [33]. Recombinant *Af*Gel1p internally cleaved a β-(1,3)-glucan (the donor) and transferred the cleaved product to the non-reducing end of another β-(1,3)-glucan (the acceptor) forming a β-(1,3)-glycosidic linkage at the transfer site. As the reaction proceeded, the elongated product itself became a donor and the reaction culminated in the formation of insoluble glucan [33]. Phr1p, Phr2p, and Gas1p proved to be endowed with the same in vitro activity as *Af*Gel1p [33]. The minimum length of the donor for Phr1p was nine glucose residues and 10 for Phr2p, Gas1p, and *Af*Gel1p. Given this type of endo/transglycosylase activity, these enzymes were collectively called β-(1,3)-glucanosyltransferases or β-(1,3)-glucan elongases [34].

GH72 enzymes are specific for β-(1,3)-glucan both as donor and as acceptor [33,35,36]. By using a laminarioligosaccharide that can only act as an acceptor (rG7) and varying the concentration of the donor substrate (rG11), the *Km* of the donor site of *Af*Gel1p was measured as 5.3 mM [37]. When substrate concentrations are low, the enzyme will utilize the hydroxyl group of water as the acceptor, resulting in hydrolysis of the glucan. In this respect, GH72 enzymes differ from the “pure” transglycosidases, e.g., plant cell wall xyloglucanosyltransferases (XET), which do not exhibit hydrolytic activity and are also catalytically more efficient [38].

**Figure 1:**
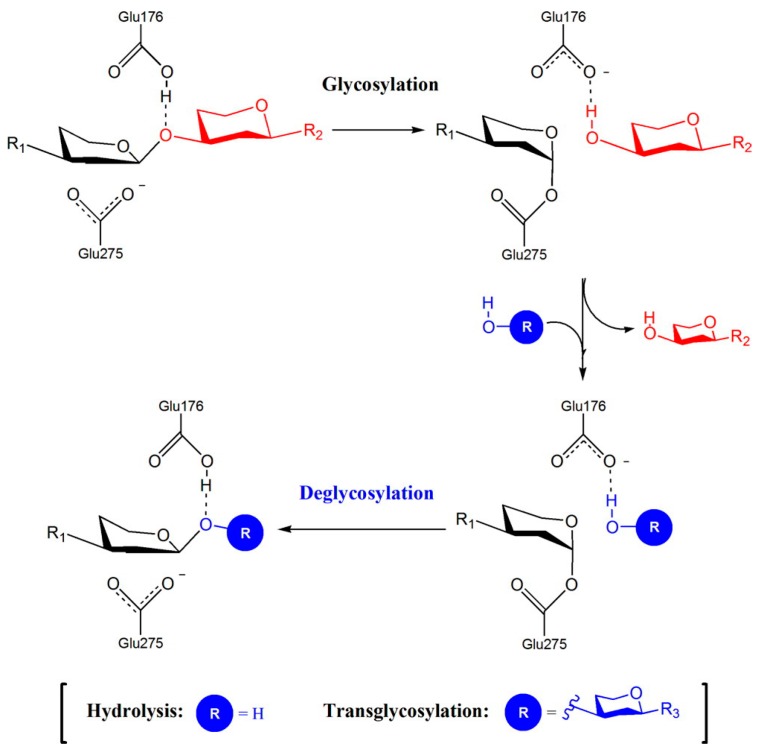
Double-displacement reaction for GHs of family 72. In the first step (glycosylation), a covalent glycosyl–enzyme intermediate (GEI) is formed and the leaving group is released. In the second step (deglycosylation), an acceptor molecule reacts with the GEI generating two different products depending on the nature of the acceptor, either a water molecule (R = H, hydrolysis product) or a sugar acceptor (R = sugar, transglycosylation product). The acid/base residue (E176) and the nucleophile group (E275) are those of Gas2p. Reprinted with permission from [39]. *Journal of the American Chemical Society*, Copyright 2016, American Chemical Society.

The reaction mechanism of GH72 enzymes is general acid–base catalysis with a proton donor and a nucleophile group. The mechanism of double displacement occurs in two steps involving the general acid/base catalyzed formation and subsequent hydrolysis (or transgycosylation) of a covalent glycosyl-enzyme intermediate (Figure 1). The –COOH group of an acid/base residue (a glutamic acid) and a dissociated carboxylate, –COO^−^, of another glutamic acid are involved. The reaction can result in an hydrolytic event if the acceptor is H_2_O or in transglycosylation if the acceptor is the non-reducing end of a laminarioligosaccharide or β-(1,3)-glucan (Figure 1). The two catalytic glutamic acid residues are in different microenvironments: the acid/base group is in a non-polar region in which the dissociation of the side chain is less favored, whereas the nucleophile group is in a polar environment that promotes dissociation.

#### 3.1.2. Domain Composition and Structure of the Phr Family of Proteins

**Figure 2:**
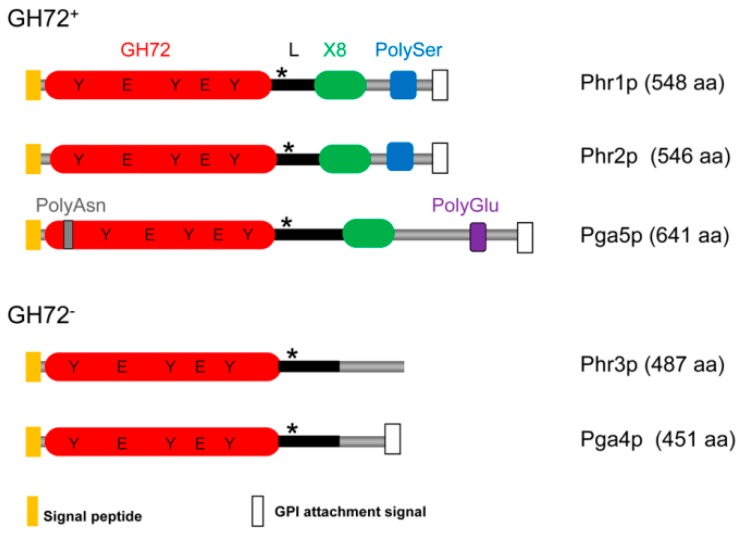
The Phr family of proteins includes members of the GH72^+^ and GH72^−^ subfamilies. The asterisk (*) indicates the conserved cysteine residue of the linker region (L). The presence of an X8/CBM43 domain defines the GH72^+^ subfamily. The catalytic glutamic acid residues and a triad of conserved tyrosines are indicated.

A diagram of the domain structure of the Phr family of proteins is depicted in Figure 2. Phr proteins share a GH72 domain including a linker region but are dissimilar in their C-terminal region. Three are members of the subfamily GH72^+^ (Phr1p, Phr2p, and Pga5p) and two of the subfamily GH72^−^ (Phr3p and Pga4p).

This division into two subfamilies derives from multiple alignments of the available GH72 sequences [27,40]. All GH72 enzymes share the GH72 domain and a linker region. The sub-family GH72^+^ contains in addition a carbohydrate binding module of family 43 (CBM43) also annotated as X8 in the Pfam database (Pfam 31.0, EMBL-European Bioinformatics Institute, Wellcome Genome Campus, Hinxton, Cambridgeshire, UK). The GH72 members that lack CBM43/X8 constitute the subfamily GH72^−^ and have a low-complexity tract in the C-terminal region.

In spite of this distinction, GH72^+^ and GH72^−^ enzymes exhibit the same in vitro transglycosidase activity, which implies a more subtle role of CBM43 in the catalytic activity of GH72^+^ enzymes (see below Section 3.1.4). In some members, a serine/threonine-rich region (Ser/Thr-box) that is highly O-mannosylated and dispensable for the activity, is present in the C-terminal tract just before the C-terminal GPI-attachment signal [41].

The GH72 domain contains two conserved glutamate residues that are essential for catalysis and three conserved tyrosine residues. Strikingly, GH72 enzymes also share a conserved pattern of regularly spaced cysteine residues that form intramolecular disulfide bonds [42]. In the GH72^+^ subfamily, two clusters of disulfide bonds are present: Cluster I comprises three disulfide bonds and involves the GH72 domain and a conserved cysteine residue of the linker region, while Cluster II comprises four disulfide bonds connecting cysteine residues located in CBM43/X8. Cluster II is absent in GH72^−^ enzymes. Extensive mutagenesis analysis and unsuccessful attempts at expressing CBM43 domain in isolation, suggested that the GH72 domain and CBM43 constitute an integrated structural and functional unit [42]. In support to this notion, the three dimensional structure of a GH72^+^ protein (Gas2p) highlighted the existence of a large interaction surface between the GH72 domain and CBM43 [43]. The GH72 domain has a (β/α)_8_ barrel structure and CBM43 is an α-helical domain. The conserved cysteine residue of the linker is sulfide-bonded to a cysteine residue of the GH72 domain, thus bringing GH72 and CBM43 domains into physical proximity. It has been shown for Gas1p that this disulfide bond is critical for the folding of the entire protein [42]. The two catalytic glutamate residues of the GH72 domain are separated by approximately 100 amino acids in the primary structure but in three-dimensional space are separated by only 5 Å, compatible with catalysis [43]. Based on crystallographic studies, a mechanism of base occlusion was proposed through which the leaving chain does not diffuse away but rather slides along the binding sites that are progressively occupied by the acceptor, limiting the access of water to the active site [43].

Replacement of either active site glutamate residue with a glutamine causes the loss of enzyme activity without affecting protein folding. Consistently, the E169Q or an E270Q mutant of Phr1p fail to complement the corresponding gene inactivation [25]. E159 and E260 are the catalytic residues in Phr2p.

**Figure 3:**
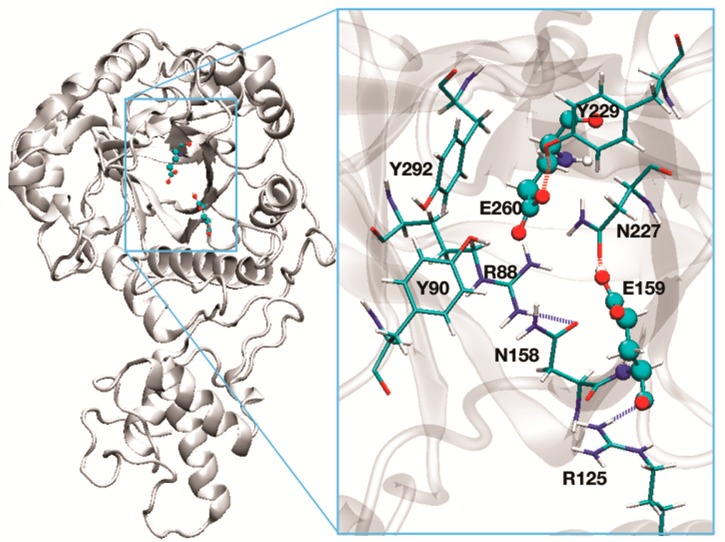
Molecular model of the three-dimensional structure of Phr2p of *C. albicans*. On the left is the full enzyme. On the right is the active site: E159, the acid/base group, and E260, the nucleophile (in ball-and-stick representation). Y90, Y292, Y229, and N227 are predicted to affect the dissociation state of the catalytic residues. R288, R125, and N158 are involved in shaping the active site pocket.

**Figure 4:**
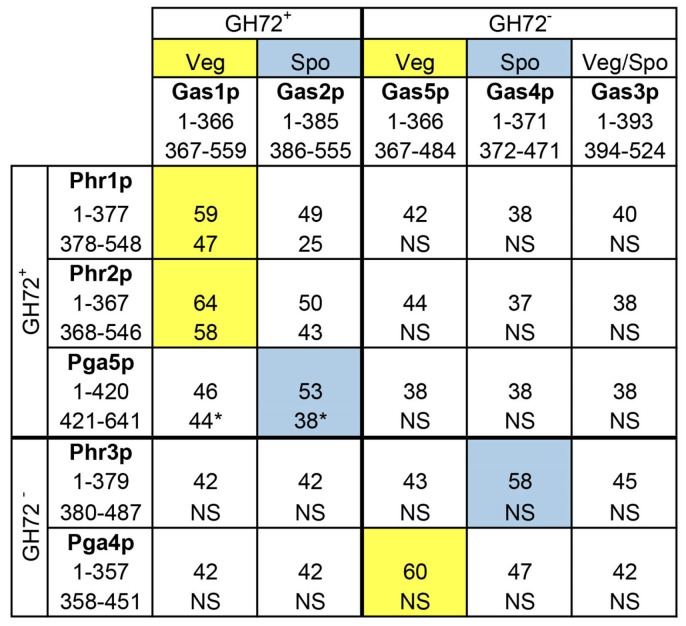
Comparison of amino acid sequences of *PHR* and *GAS* families. Colored blocks indicate *C. albicans* proteins most closely related to functionally defined *S. cerevisiae* proteins. Yellow: proteins active in cell wall assembly during vegetative growth of *S. cerevisiae.* Light blue: proteins active in spore wall assembly in *S. cerevisiae*. * indicates the presence of a Poly(Glu) tract in the Pga5p C-terminal region.

Given the high homology between Phr1-Phr2p and Gas2p (see Section 3.1.3, Figure 4), a molecular model of both proteins was built (see Section 3.1.3). Superimposition of the structures indicated that the stereochemistry of the active site is identical for Phr1p and Phr2p. In Figure 3, the predicted structure of the active site of Phr2p is shown. The catalytic residues are separated by 5 Å and a triad of tyrosines (Y90-Y229-Y292) and N227 surround these residues. Residue Y229, being less than 2 Å from E260, is predicted to greatly influence the dissociation state of its carboxylate. This tyrosine triad and N227 likely create a network of H-bonds and a movement of protons responsible for the protonated state of the catalytic glutamic acid residues. Indeed, replacement of individual tyrosine residues with Phe or Gln in equivalent positions of Gel2p has been shown to affect the transglycosylation reaction [43]. Other residues of Phr2p, R88, R125, and N158 line the active site and are predicted to be involved in substrate binding.

#### 3.1.3. Active, Inactive, and Anomalous Members of the Phr Family of Proteins

Given the medical importance of *C. albicans* as a fungal pathogen, the redundancy of Phr proteins was explored more deeply to gain information on the activity of the proteins. First, the amino acid sequences of the GH72-Linker domain and of the C-terminal region were compared to the respective sequences of the Gas family of proteins (Figure 4) [36]. As shown in Figure 4, beside the high degree of amino acid identity of Phr1p and Phr2p to Gas1 protein, Pga4p showed a high degree of identity to Gas5p (60%) in the first 360 amino acids (GH72 domain+ linker). Gas1p and Gas5p are expressed during vegetative growth in yeast and are required for cell wall formation with a predominant expression and function of Gas1p over Gas5p [44]. Interestingly, Phr3p and Pga5p are more closely related to the sporulation-specific Gas1-homologs Gas4p and Gas2p, respectively. The Gas2–Gas4 protein pair is specialized in spore-wall formation in yeast [45]. However, *C. albicans* does not undergo the process of meiosis and sporulation. This suggests that Phr3p and Pga5p, if active, have probably been directed to other processes. However, Phr3p and Pga5p contain various anomalies. The CDS of *PHR3* has an unbiased use of codons, an index of low expression and Phr3p lacks an apparent GPI attachment signal. Pga5p has (i) a stretch of 10 consecutive Asn residues (polyAsn, encoded by the cognate codon AAC) and (ii) a stretch of 12 glutamic acid residues (encoded by 10 GAA and two GAG codons). In addition, as mentioned above, *PHR3* and *PGA5* transcripts are of low abundance.

Furthermore, no phenotypic defects have been associated with deletion of *PHR3* or *PGA5* (see further Section 4) [27]. Thus, *PHR3* and *PGA5* appear to be genetically inactive member of the *PHR* family.

With reference to phylogenetic relationships, *PHR1* and *PHR2* are orthologs of *GAS1* (clade G1), *PGA4* is the ortholog of *GAS5* (clade alpha), *PGA5* is the ortholog of *GAS2* (clade G2) and *PHR3* is an ortholog of *GAS4* (clade gamma) according to a recent evolutionary analysis [27].

Therefore, recent studies on the catalytic activity of the Phr proteins concentrated on Phr1p, Phr2p and Pga4p. By use of a new fluorescent enzyme microassay, designed in collaboration with Vladimir Farkas of the Laboratory of Glycomics, the basic catalytic properties of recombinant Phr1p, Phr2p, and Pga4p were investigated [36]. Surprisingly, no activity was detected for recombinant Pga4p. Therefore, out of the five members of the Phr family of *C. albicans*, so far only two genes appear to encode active GH72 isoenzymes (*PHR1* and *PHR2*) and one gene (*PGA4*) likely encodes a structural mannoprotein. This is different from the situation in *S. cerevisiae* where, out of five Gas proteins, four are catalytically active and only one does not show activity (Gas3p) [40]. Table 1 summarizes the results of studies on the catalytic properties of the Phr1 and Phr2 proteins.

The retention of only two enzymatically active GH72 isoenzymes in *C. albicans* may be linked to the commensalism of this opportunistic fungal pathogen that has the human body as a unique host and also to the lack of complex differentiation pathways such as meiosis and sporulation. The human fungal pathogen *A. fumigatus* is a saprophyte exposed to the natural environment (soil) and spores must be inhaled to produce invasive infections in immunocompromised patients. Moreover, the *A. fumigatus* life cycle is very complex. This may justify the presence of seven GH72 isoenzymes in this human fungal pathogen.

Interestingly, the gene family encoding catalytic subunits of β-(1,3)-glucan synthase (*FKS/GLS*) show a similar situation. In *S. cerevisiae*, three *FKS* genes are functional. The genome of *C. albicans* contains three *FKS* homologs but only one of them, *GSC1*, is essential, whereas *GLS1* encodes a truncated protein and *GSL2* expression, if any, is very low. This proved useful in finding inhibitors of β-(1,3)-glucan synthesis that kill *C. albicans*.

#### 3.1.4. β-(1,3)-glucan Elongation and Branching: Cooperation between Family GH72^+^ (Gas/Phr/*Af*Gel) and Family GH17 (Bgl/Bgt) Transglycosylases

Although it has long been known that the glucan network is made of branched β-(1,3)-glucan fibers and that lateral branches create anchoring sites for other cell wall components (β-(1,6)-glucan, chitin and mannoproteins), the identification of the branching activities has been a challenge for fungal molecular biologists for many years. A transglycosylase capable of adding β-(1,6)-branches to β-(1,3)-glucan, Bgl2p, was identified in *S. cerevisiae*, *C. albicans*, and *A. fumigatus* (*Af*Bgt1p and *Af*Bgt2p), but the role of these proteins seemed negligible since the corresponding deletion strains did not manifest any remarkable phenotypic trait except higher sensitivity to the chitin synthesis inhibitor Nikkomycin Z and a lower kinetics of biofilm formation of the *C. albicans bgl2* null mutant [46,47,48].

Yeast Bgl2p is a 35-kDa glycoprotein of the GH17 family, localized to the cell wall and also secreted in the medium. In vitro Bgl2p/*Af*Bgt1p catalyze the transfer of a disaccharide unit (laminariobiose) from the reducing end of a laminarioligosaccharide (≥ 5 glucose units) to the non-reducing end of an acceptor laminarioligosaccharide (≥4 glucose residues) creating a β-(1,6)-glycosidic linkage at the transfer site [49]. The resulting oligomer is a “kinked” linear β-(1,3)-glucan. Interestingly, another Bgl2-homologous protein identified in *A. fumigatus*, *Af*Bgt2p, is able to catalyze the transfer of the glucan chain to an internal glucose. Since the internal glucose is linked to the neighboring glucose units by its C-1 and C-3 and to the C-1 of the transferred glucose/glucan chain by its C-6, this activity creates a so-called “branching point”.

Recently, a relevant advance in the field was the finding that (i) GH72 enzymes are endowed with not only elongation, but also branching activity, (ii) GH72 enzymes cooperate with the Bgl/Bgt GH17 proteins in creating the branched core glucan and (iii) the β-(1,3)-glucan of a *gas1*Δ*bgl2*Δ mutant is devoid of β-(1,6)-branching and exhibits a synthetic sick phenotype [50].

The amount of branching in the cell wall is comparable in yeast, *A. fumigatus* and *C. albicans*. The proposed model predicts that Gas1p forms branches either by introducing a β-(1,6)-linkage on an internal glucose unit of a self-created β-(1,3)-linked long glucan or alternatively by catalyzing the transfer of a β-(1,3)-glucan to a C-3 on a kinked linear β-(1,3)-glucan created by Bgl2p. Thus, Gas1p and Bgl2p cooperate in elongation and branching of the cell wall glucan. Bgl2p preferentially uses short chains (≥5 glucose residues) whereas the branching activity of Gas1p first requires the formation of long glucans (≥11). The contribution of Gas1p and Bgl2p in the creation of β-(1,6)-linkages on β-(1,3)-glucan was determined by analyzing the percentage of branching in the deletion mutants. While the *bgl2*Δ mutant showed a 15% decrease in branching and the *gas1*Δ mutant exhibited a 70% decrease, the double *gas1*Δ *bgl2*Δ mutant had no branching and manifested a synthetic sick phenotype characterized by a slow growth rate, round cell shape, random budding pattern and increased sensitivity to cell wall perturbing agents compared to the wild type or the single *bgl2*Δ or *gas1*Δ [50]. Thus, GH72 enzymes represent the major glucan elongation and branching activity and cooperate with GH17 enzymes. No *bgl2*Δ *phr1*Δ or *bgl2*Δ *phr2*Δ double deletions have been described in *C. albicans*.

Interestingly only GH72^+^ proteins (Gas1p, Gas2p, and *Af*Gel4p) proved to be endowed with elongation and branching activity, whereas GH72^−^ enzymes (Gas5p, *Af*Gel1p, and *Af*Gel2p) exhibited the elongation but not the branching activity. This result suggests that CBM43 is essential for proper positioning of the substrate required for the branching activity. This notion provides the first functional distinction between the two sub-families.

**Figure 5:**
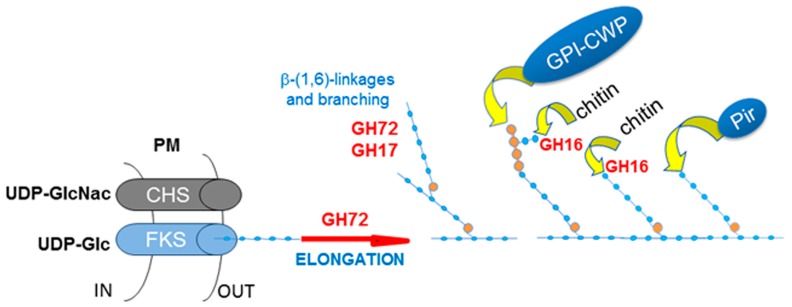
Scheme for the assembly of cell wall components in the extracellular space. In red are indicated the family of transglycosylases described in the text; in light blue: β-(1,3)-glucan; in orange β-(1,6)-glucose-linked residues. GH72 and GH17 cooperate in creating the core glucan structure and the anchoring sites for the other cell wall components. Pir are proteins with internal repeats that are directly linked to β-(1,3)-glucan chains through alkali labile ester linkages [52].

In conclusion, the GH72 enzymes work in concert with the plasma membrane β-(1,3)-glucan synthase complex, which synthesizes short chains using Uridine diphosphate glucose (UDP-glucose) as a substrate and extrudes the polymer into the periplasmic space. GH72 enzymes then process these short β-(1,3)-glucan chains in the extracellular space. Given their in vitro activity, the current proposed model predicts a role of GH72 enzymes in creating long glucan chains and in elongating branching points [50]. Figure 5 summarizes the main steps of cell wall assembly.

The in vivo impact of GH72 enzymes on cell wall β-(1,3)-glucan was demonstrated by pulse-chase experiments in *C. albicans* cells [25] (see Section 4). Another example of the impact of the elongation activity on cell wall assembly in vivo came in 2012. Cabib showed that the lack of Gas1p converts yeast HMW β-(1,3)-glucan into a polydisperse glucan [51]. Studies of *A. fumigatus* have also demonstrated the in vivo effects [50].

### 3.2. Where and When Do Phr Proteins Play Their Biological Role in *C. albicans* Cells?

An important piece in the puzzle of defining the biological role of a protein is its localization. With respect to Phr1, Phr2, and Pga4 proteins, modification by GPI attachment affects their localization. This modification occurs in the ER and, together with *N*-glycosylation, promotes the transport of proteins through the secretory pathway to cell surface. The GPI moiety is a means of anchoring extracellular proteins to the outer layer of the plasma membrane and also confers lateral mobility to the proteins. In addition, in the majority of Ascomycetes, the glycan moiety of the glycolipid can be processed at the cell surface by a putative transglycosidase that transfers the lipid-less portion of GPI (GPI-remnant) to the cell wall polysaccharide network and consequently covalently cross-links the protein to the cell wall. In this way, a plasma membrane GPI protein is converted into a cell wall protein (CWP), termed GPI-CWP. In *C. albicans*, the majority of the CWPs are GPI-CWPs and play a crucial role in fitness and virulence [53]. In general, only a minor fraction of GPI proteins are transformed into GPI-CWPs, whereas the majority remain attached to the plasma membrane. However, the proportion of these fractions varies from protein to protein.

Thus, it is not surprising that MS analyses of the *C. albicans* cell wall proteome identified Phr1p, Phr2p, and Pga4p as GPI-CWPs [24,54,55]. Moreover, in a proteomic study of the cell surface, Phr1p and Pga4p were detected in the material shaved from intact *C. albicans* cells, indicating their surface location [56]. It is still debated whether the GPI proteins endowed with enzymatic activity retain their activity when they are covalently linked to the cell wall. The GPI remnant is connected to a β-(1,6)-glucan chain and this in turn is linked to β-(1,3)-glucan [53]. The short β-(1,6)-glucan may provide a flexible arm that lets the enzyme operate within a substantial radius of action and at more superficial sites of the cell wall. However, this attractive hypothesis awaits experimental demonstration.

**Figure 6:**
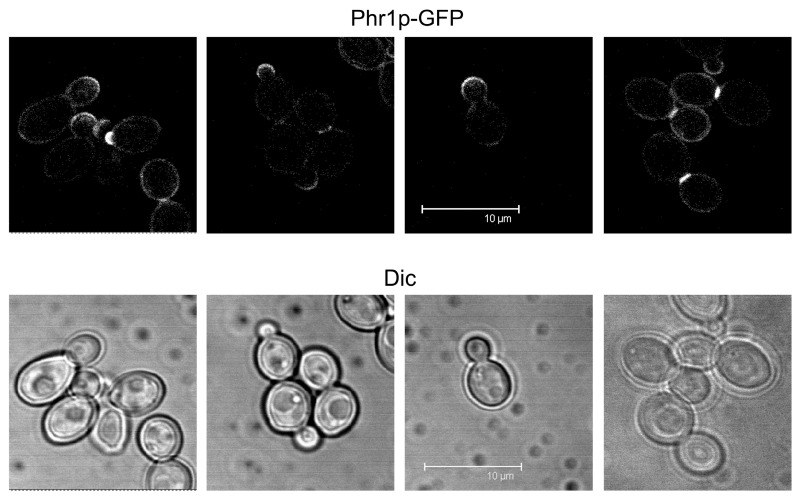
Localization of Phr1p-GFP in *C. albicans*. Cells growing in YPD-150 mM HEPES pH 7.5 at 25 °C.

The localization of Phr1p was investigated in detail using an internal GFP tag [57]. In vegetative growth, Phr1p-GFP localized to the presumptive bud site and to the bud periphery, sites where the cell wall is more plastic and new material is incorporated during the polarized growth phase of the cell cycle (Figure 6). During the isotropic growth phase and in particular in cells at cytokinesis, Phr1p-GFP is concentrated at the septum, where it forms a brightly fluorescent band (Figure 6), suggesting a role in the assembly of the secondary septa.

**Figure 7:**
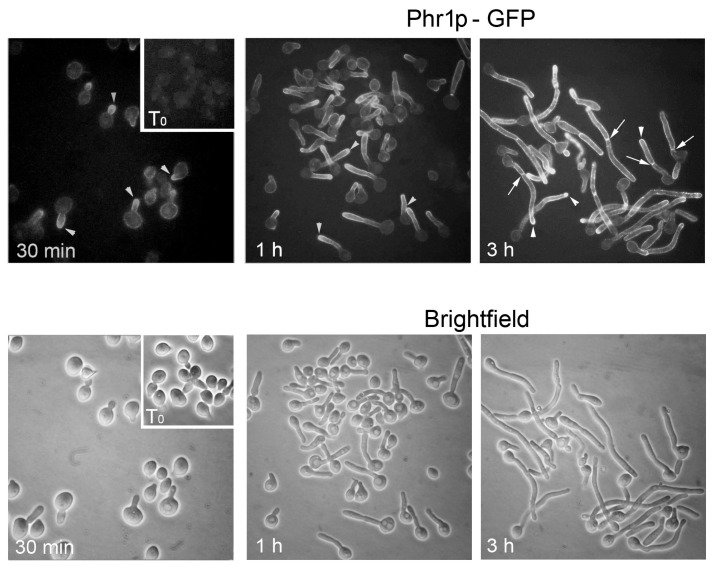
Localization of Phr1p-GFP in *C. albicans*. Phr1p-GFP localization in conditions of induction of hyphal growth in M199–150 mM HEPES, pH 7.5 at 37 °C, Bar: 5 µm. Modified from [57].

When *C. albicans* is induced to develop hyphae, Phr1p-GFP localizes to the site of germ tube emergence, to the tip of the germ tube, and to the septum (Figure 7). Later, it localizes to the hyphal apex and also distributes along the lateral walls of the hyphae.

Localization at the site of germ tube emergence, the germ tube tip and the hypha apex, and lateral hyphal walls is dependent on the actin cytoskeleton, whereas microtubules control the localization of Phr1p at the septum [57]

Given that the Phr proteins are located in both the cell membrane and the cell wall, covalent cross-linking to the cell wall might be expected to sequester the protein from membrane turnover. We have previously shown that is possible to specifically localize a GPI-CWP under conditions that induce the degradation of the plasma membrane form [58]. Preliminary experiments indicate that during a shift from pH 7.5 to 4.5, a condition that switches off the expression of *PHR1*, Phr1p-GFP totally disappears in a couple of hours from the cell contour (Popolo and Degani, unpublished data). This suggests that all of the Phr1p-GFP is subjected to protein turnover and no covalently cross-linked cell wall forms are detectable. This differs from Gas1p-GFP in yeast, which was shown to be cross-linked to the wall of the bud scars, where brightly fluorescent rings overlapped with the chitin rings on the cell surface [29].

Finally, the recruitment of Phr1p-GFP to hypha-damaged sites caused by neutrophil attack in vitro led to the proposal of a potential repair role of Phr1p [59]. This suggests that the synthesis of a new wall induces a lateral mobility of pre-existing Phr1p molecules to the damaged site or that more molecules are recycled by endocytosis and trafficking to the plasma membrane upon injury of subapical hyphal compartments.

## 4. *PHR* Family in Morphogenesis and Virulence

*PHR1* and *PHR2* are the most thoroughly studied members of the *PHR* family and exhibit the more overt mutant phenotypes. Cell morphology is dramatically affected in *phr1* and *phr2* deletion mutants [14,16]. In accord with their transcriptional patterns, the morphological deficits are pH-conditional. A *phr1* null mutant grows with a normal budding pattern and typical ellipsoidal yeast shape in acidic culture conditions. At pH 8, 37° C, conditions that promote germ tube formation, the mutant fails to form germ tubes and instead forms rounded outgrowths with extremely wide septa [14]. At 25° C, which promotes growth as yeast, the initial daughter cells of the mutant have a flattened appearance, more wide than long, with wide bud necks [14]. After several generations at alkaline pH, at either temperature, the mutant forms aggregates of enlarged, rounded cells with wide bud necks [14], a phenotype similar to that of *S. cerevisiae Gas1* mutants [60]. Mutants lacking *PHR2* show similar morphological abnormalities, but the defects appear under acidic culture conditions [16]. One notable difference between *PHR1* and *PHR2* mutants is that *phr1* null mutants continue to grow at alkaline pH, albeit with a reduced growth rate, but *phr2* deletion mutants cease growth within two generations at acidic pH [16]. The essential nature of *PHR2* under acidic growth conditions was also observed in tetracycline-conditional expression mutants of *PHR2* [61]. Despite this difference, forced expression of *PHR1* complements the morphological and growth defects of a *phr2* mutant and vice versa, demonstrating that they encode analogous activities [16].

The morphological changes in the mutants are accompanied by biochemical changes in the cell wall. When cultured at alkaline pH, the cell wall glucan content of a *phr1* null mutant is either unaltered [25,62] or reduced by about 25% [63], depending perhaps on the method of analysis. However, the alkali-soluble fraction is increased [25,62], suggesting a defect in proper cross-linking of nascent β-(1,3)-glucans and consistent with the in vitro enzymatic activities. At the same time, there is a 5- to 6-fold increase in the amount of zymolase-insoluble glucan, β-(1,6)-glucan, cross-linked to chitin, and a similar increase in chitin content [25,62], further indicating a deficiency in β-(1,3)-glucan processing. In line with these changes, a *phr1* mutant is more sensitive to calcofluor white, nikkomycin Z, and SDS, but more resistant to caspofungin [62,63]. Analysis of the cell wall of *phr2* mutants is more limited, but a similar accumulation of chitin-linked β-(1,6)-glucan occurs at the restrictive pH [25]. These biochemical changes manifest as ultrastructural alterations in the cell wall of both yeast and hyphae [63,64].

Given the impact of *PHR1* and *PHR2* on the cell wall and the importance of this structure in mediating host–pathogen interactions, loss of *PHR1* or *PHR2* compromises virulence of *C. albicans*. Moreover, the differential expression of these two genes results in niche-specific attenuation of virulence. *PHR1* null mutants are avirulent in a mouse model of systemic infection employing either BALB/c or CD2F1 mice [65,66]. Although initial colonization of brain, liver, and kidney tissues is comparable to wild-type cells, subsequent proliferation of the mutant is greatly reduced and histological sections demonstrate a lack of hyphae and the presence of enlarged rounded cells similar to those seen in vitro [65,66]. However, in a rat model of vaginal candidiasis, a *phr1* null mutant is fully virulent [65]. In contrast, deletion of *PHR2* imparts the inverse virulence phenotypes. The *phr2* null mutant is fully virulent in the systemic model, but is strongly attenuated in the vaginitis model [65].

Results of the systemic model correlate well with the pH-dependent expression pattern of these genes, given the slightly alkaline pH (7.3) of blood and tissues and the alkaline-induced expression of *PHR1*. Indeed, expression of *PHR1* has been demonstrated in vitro in blood and in vivo in various tissues and infection models including samples from patients with oropharyngeal candidiasis [67] and a mouse model of oropharyngeal candidiasis [68], during intraperitoneal infection and liver invasion [69], in infected rabbit kidneys [70], in the cecum during intestinal colonization of mice [71], and in a zebrafish infection model [72]. Results of the vaginitis model are less readily explained as the reported pH of the rat vagina averages around 6.95 [73]. Acidification of the vaginal environment during infection might account for the *PHR2* requirement or other environmental variables of the vagina might influence *PHR2* expression.

The virulence attributes impacted by *PHR1* and *PHR2* are not completely defined, but include adhesion, immune system interactions, and biofilm formation. Cells lacking *PHR1* are unable to penetrate or invade reconstituted human epithelia, which correlates with the mutant’s inability to form hyphae and a gross reduction in adherence to epithelial cells and polystyrene [74]. The adherence defect may reflect altered expression of adhesion proteins or failure to properly incorporate them into the cell wall. It is known that the architecture of cell surface mannoproteins is disrupted in *phr2* mutants as evidenced by the aberrant exposure of the glucan layer in these cells [75]. Exposure of the glucan layer may contribute to the loss of virulence of *PHR* null mutants as the mannan layer appears to shield the cell’s glucan layer from immune detection via Dectin-1 and the ensuing inflammatory response [75,76].

The formation of biofilms, a matrix-embedded community of cells, plays a significant role in the biology and pathogenicity of *C. albicans* [77]. An important component of the biofilm matrix is β-(1,3)-glucan [78]. Expression of *PHR1* is increased in biofilm versus planktonic cells [79,80] and cells lacking *PHR1* produce nearly 10-fold less matrix glucan [81]. In contrast, another study showed that overexpression of *PHR2*, but not *PHR1*, enhanced biofilm occupancy, but not adherence or biofilm mass [82]. These differences likely reflect variations in media composition and pH used for biofilm formation but nonetheless implicate these enzymes in the process. A further indicator is the reduced expression of *PHR1* when biofilm formation is blocked by mutation of *SUN41* [83].

Another biologically important role of biofilms is their implication in mating. *C. albicans* cells are typically heterozygous a/α at the mating-type locus. Cells that become homozygous a/a or α/α can undergo a reversible switch between white-phase and opaque-phase cells, which differ in size, morphology, and virulence properties [84]. Opaque cells are competent for mating and α-factor, the pheromone secreted by α/α cells, promotes biofilm formation by white cells in the population [85]. The pheromone response entails upregulation of a number of genes encoding cell wall proteins including *PHR1* and *PHR2*, both of which contain a white pheromone-regulated element (WPRE) within their promoter regions [85]. Furthermore, the switching process itself, from white to opaque, is sensitive to pH [86]. Deletion of *PHR1* did not prevent the pH effect, but *PHR2* was required to maintain cells in the opaque phase at acidic pH [86].

In contrast to *PHR1* and *PHR2*, few functional studies have been conducted for *PHR3*, *PGA4*, or *PGA5*. Mutants lacking *PHR3*, *PGA4*, or *PGA5* show no significant alterations in growth, morphology, or virulence [17,87]. Nor do the mutations alter sensitivity to cell wall perturbing agents such as calcofluor white, Congo red, or SDS [17]. Deletion of *PGA4* does, however, appear to influence cell wall structure as the mutant has enhanced resistance to caspofungin and anidulofungin, antifungals that inhibit glucan synthase [63]. Furthermore, the mutant has a slightly reduced content of β-(1,3)-glucan in the cell wall and is more sensitive to osmotic stress when cultured with glucose vs. lactose as the carbon source [63,88,89]. Expression of *PGA4* is downregulated in *ACE2* mutants, which are defective in filamentation under hypoxic conditions [90]. The only phenotype reported for a *PHR3* mutant is a reduction in agar invasion ability in a heterozygous transposon insertion mutant [91]. No phenotypes have been associated with mutation of *PGA5*.

## 5. The Adaptive Response to Cell Wall Stress Induced by the Lack of Phr1p

### 5.1. The Response to Cell Wall Stress

Among the stress conditions encountered by *C. albicans,* immune insults or drug-induced damage can harm cell wall integrity. In general, the response to stress involves a sensing apparatus that activates signaling pathways and conveys/amplifies the input signal. The cellular changes range from gene expression to chromatin modifications, from regulation of protein activity by post-translational modifications to degradation of selected proteins. The combination of these responses induces an adaptation of the cell to the new condition and the consequent downregulation of the signaling pathways.

**Figure 8:**
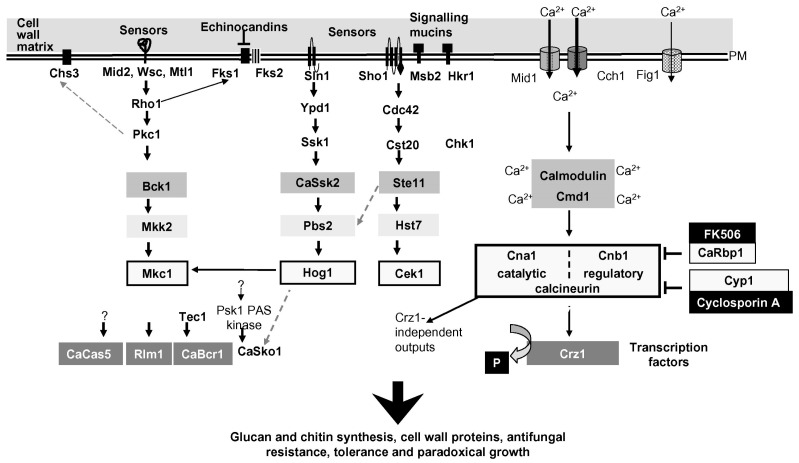
Diagram of input signal and signaling pathways activated in *C. albicans* by cell wall stress. On the right (in the black box), the inhibitors of calcineurin and in white the target proteins. P in the black box represents the phosphate group that calcineurin removes from Crz1p. The question mark on Psk1p indicates that cell wall damage activates Psk1p in an unknown manner. The question mark on CaCas5 indicates an unknown activator of this TF that regulates the cell wall stress-response. Reproduced from [92].

The response to cell wall stress conditions has been intensely studied both in *S. cerevisiae* and in *C. albicans* using different “stressors” such as mutations in cell-wall-related genes, inhibitors of β-(1,3)-glucan synthesis (echinocandin, caspofungin, and mycofungin), cell wall perturbing agents [Calcofluor, a dye that binds nascent chitin chains or Congo red, a compound that binds β-(1,3)-glucan] or treatments with β-(1,3)-glucanases that hydrolyze the cell wall glucan (e.g., Zymolyase). Most of these stressors can be used either in acute or chronic administration. Other widely used stressors are SDS or temperature, although they act indirectly. According to the type of stressor, the activation of signaling pathways can be different and vary in kinetics. In this section, we will limit discussion to the cell wall stress generated by alterations of β-(1,3)-glucan synthesis or assembly (absence of Phr1p). Figure 8 recapitulates the signaling pathways of cell wall stress from the surface to the effectors.

Loss of the mechanical resistance of the cell wall is a relevant stress signal. The associated stretching or warping of the plasma membrane alters the intracellular concentration of Ca^++^, a crucial second messenger in eukaryotes. This occurs through the action of mechanosensitive-membrane channels (Figure 8) or release of Ca^++^ from intracellular stores such as that of the ER, an exquisite sensor of cell stress in eukaryotes, or vacuoles [93] (not shown in Figure 8). Specific sensors have been identified as belonging to the Wscp/Mid/Mtl families of type I transmembrane glycoproteins. These proteins have an integrin-like structural organization and detect alterations in chemical/physical properties of the cell wall through their ectodomain. Similar to Wsc proteins, mucin-like proteins or other highly glycosylated surface proteins also contribute to monitoring the status of the cell wall.

The cell wall stress response appears to be common, at least in part, to other yeast and human pathogenic fungi and involves evolutionarily conserved modules of Mitogen-Activated Protein (MAP) kinases comprised of a MAP kinase kinase kinase (MKKK) and a MAP kinase kinase (MKK) that catalyzes the dual phosphorylation of a MAPK, which in turn phosphorylates crucial substrates [94]. In *C. albicans*, the key MAPK sensitive to cell wall stress is Mkc1p, a homolog of Mpk1p/Slt2p of *S. cerevisiae*. Rho1p, a small GTPase, activates Protein kinase C (Pkc1p), an evolutionarily conserved Ser/Thr protein kinase that is essential for preventing cell lysis in yeast and regulates Fks1p, the catalytic subunit of glucan synthase.

In *C. albicans*, the PKC1-Mkc1p pathway, also called the PKC cell wall integrity (CWI) pathway, is transiently activated by hypo-osmotic shock, cell wall stress, oxidative stress, and physical contact [95,96]. In cell wall stress, the transcriptional response effected by Mkc1p is dominated by the activation of the transcription factor (TF) Rlm1p. In addition, the MAP kinases Cek1p, required for filamentation, and Hog1p, typically activated by high osmolarity, oxidative stress, and clamydospore formation, are both triggered [96,97]. Finally, through binding of calcium to calmodulin, activation of the calcineurin/Crz1p pathway leads to dephosphorylation of a latent TF, Crz1p, which translocates from cytoplasm to the nucleus and activates transcription [98]. Recent work in yeast revealed the involvement of the cAMP-dependent protein kinase pathway (cAMP-PKA), typically implicated in nutritional signaling in yeast and essential for viability. Loss of Gas1p function or treatment with caspofungin induces the CWI pathway but represses the cAMP-PKA pathway [99,100]. Inhibition of PKA signaling induces a decrease in telomeric silencing and an increase in rDNA silencing, both changes mediated by Sir2p, a NAD^+^-dependent histone deacetylase and a subunit of the rDNA silencing complex RENT [100]. Whereas it is unquestionable that the lack of Gas1p or treatments with caspofungin can cause important changes in chromatin silencing [100,101,102], it seems difficult to reconcile the involvement of nuclear Gas1p molecules in the direct control of Sir2p-mediated transcriptional silencing, as was proposed by some authors [103]. Several lines of evidence argue against this: first, Gas1p was never detected in the nucleus using internally tagged GFP fusions versus C-terminal fusions at the GPI-anchor signal, second, the lack of biochemical evidence that Gas1p can transfer a β-(1,3)-glucan to a protein (Sir2p) and third, the chance of fortuitous in vitro interaction between Gas1p domains expressed in bacteria, an unsuitable host for folding of GH72 enzymes, and Sir2p.

Repression of the cAMP-PKA pathway also triggers the accumulation of the TFs Msn2p/Msn4p in the nucleus. In yeast, Msn2p/Msn4p bind the STRE element that is present in the promoters of a set of genes regulated by cell wall stress [104]. A recent link with transcriptional silencing was evidenced by the presence of a STRE element in *PNC1*, encoding a nicotinaminidase, which provides a connection between the level of nicotinamide and inhibition of Sir2p [100]. Regarding the cAMP-PKA pathway in *C. albicans*, it is essential for the dimorphic transition, but not for growth. No data are available on the potential repression of the cAMP-PKA pathway in response to cell wall stress in *C. albicans*. In addition to these pathways, studies of cell wall stress induced by caspofungin treatment recently revealed that phosphatidylinositol(4,5)-bisphosphate and the septin ring also play a role in cell wall integrity [105].

### 5.2. The Adaptive Response in *C. albicans* Cells Lacking Phr1p: Protecting Cell Integrity by Preventing Cell Polarization

**Figure 9:**
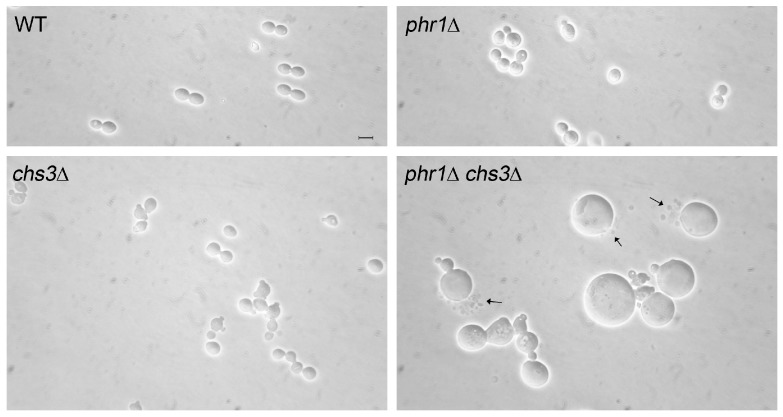
Phenotype of the indicated mutants in liquid YPD-150 mM HEPES, pH 8 at 25 °C (vegetative growth). Cells were sonicated for three cycles of 6 s. The percentages of lysed cells were: WT: 2%, *phr1*∆: 14%, *chs3*∆: 55% and *phr1*∆ *chs3*∆: 85%. The arrows indicate fractures of the cell wall and release of cellular material, Bar: 5 µm. Unpublished results.

Our interest in GH72 enzymes motivated a study on the cell wall stress generated by the lack of this activity in *C. albicans*. The pH-conditional phenotype of *phr1*∆ cells offered the advantage of simply switching on cell wall stress by a pH up-shift. As described in Section 4, the round cell shape and the widening of the mother–daughter neck of *phr1*∆ cells reflect the severe weakening of the cell wall that does not efficiently counteract the expanding force caused by the high intracellular turgor pressure, similarly to the condition of hypo-osmotic shock. As expected from the well-known phenomenon of Chs3p-mediated hyper-accumulation of chitin in response to cell wall stress, a mutant deficient in *PHR1* and *CHS3* showed abnormal enlargement and extensive lysis when cultured as yeast at alkaline pH at 25 °C (Figure 9). The stretching resistance of chitin fibrils likely creates a mechanical barrier to the expanding force coming from inside [34]. The other chitin synthases had no significant effect on morphology or viability in this condition.

We characterized the genome-wide transcriptional profile of *phr1*Δ cells during hyphal development because this is the stage of maximum adherence to substrates and capacity for penetration of tissues. After the induction of hyphal growth, in the wild-type cells Mkc1p, Cek1p, and Hog1p were transiently activated, whereas in a *phr1*∆ mutant these MAP kinases remained persistently hyper-stimulated for at least 7 h, indicative of the severe cell wall stress and defective morphogenesis experienced by *phr1*∆ cells [106].

Three different time points, 1, 3, and 5 h after induction of hyphal growth, were examined for transcriptome analysis [106]. No transcriptionally modulated cross-compensation by the other *PHR* paralogs was detected. The functional categories of genes more transcribed in the mutant than in the wild type and those more repressed in the mutant than in the wild type were many. Changes in the “Cell wall” category were the most relevant at all three time points. Genes in the functional category of “RNA and ribosome” maturation were among the most represented repressed genes at 1 h, and this is probably related to the abovementioned increase in silencing of rDNA.

The “Cell wall” category included:

- a set of genes encoding mannoproteins *PGA23*, orf19.750, *RBR1*, *PGA13*, *PGA54*, *RBT4*, *ECM331* and *PGA6*, suggesting a qualitative and quantitative change in the pattern of cell wall mannoproteins.
- *CHS2* and *CHS8* encoding the Class I Chitin synthases, Chs2p and Chs8p, and reported to be subject to Mkc1p and Hog1p-mediated transcriptional increase in the presence of cell wall stress.
- *CHS7* encoding the ER-chaperone for Chitin synthase 3 (Chs3p), a post-translational regulated Class IV Chitin synthase.
- *CRH11*, a homolog of *S. cerevisiae CRH1* and a member of *C. albicans* family GH16 trans-glycosylases together with *UTR2*/*CSF4* and *CRH12*. GH16 proteins cross-link chitin with glucans by transferring the reducing end of chitin to either the non-reducing end of a β-(1,3)-glucan chain or to a β-(1,3)-glucose side-branch of a β-(1,6)-glucan chain [107] (Figure 5).

The existence of an additional Class I chitin synthase in *C. albicans*, *CHS8*, stimulated our interest in the physiological role of chitin synthases in the response to CW stress. This was examined in a set of double mutants lacking *PHR1* and one or more of the chitin synthase genes. Here we summarize the most relevant findings. The combined deletion of *PHR1* and inactivation of a *CHS*X (X = *CHS2*, *CHS8* or *CHS2*, *CHS8*) gene was examined during filamentous growth. The observed phenotypes depended on ambient pH and the composition and type of medium (liquid or solid). In M199, at the less restrictive pH (pH 7.5) at 37 °C, only *phr1*∆ *chs3*∆ cells displayed a worsening of the *phr1*∆ mutant phenotype with progressive swelling and cell lysis. At the more restrictive pH 8, *phr1*∆ *chs3*∆ cells were highly aggregated and, after sonication, appeared arrested at the onset or early germ tube emergence with a majority of lysed cells. Although Chs2p and Chs8p contributed to the chitin increase in the *phr1*∆ mutant, they were not crucial for viability [106].

On M199 agar-solidified plates the *phr1*∆ *chs3*∆ mutant did not geminate at either pH 7.5 or pH 8 and the phenotype was not by-passed by inclusion of 0.8 M sorbitol in the plates. The parental strains were unaffected in their capacity to germinate and formed colonies. On the same medium, at pH 8 only, the *phr1*∆ *chs8*∆ mutant also displayed an interesting phenotype. The double mutant germinated but cells did not progress further giving rise to abortive microcolonies visible only under the stereomicroscope. This phenotype was not sorbitol-remediable. No effects of the *CHSX* gene deletions were visible in the wild-type background at pH 8.

**Figure 10:**
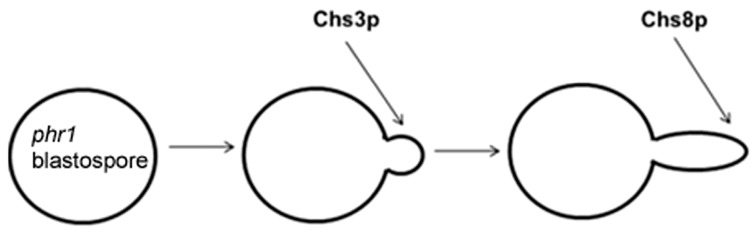
Model of the sequential involvement of the chitin synthases in the adaptation to cell wall stress of a *phr1* cell during induction of hyphal growth in M199 at a restrictive pH. First, at the time of germ tube emergence Chs3p is crucial in liquid media and, if not present, *phr1*Δ cells arrest at early germ tube emergence, swell and die. On solid surface, Chs3p is crucial for the onset of germination and if absent *phr1*Δ cells do not produce colonies. Chs8p is crucial for the development of the germ tube. Once the germ tube has emerged, Chs8p acts to protect the tip. If Chs8p is absent during growth on a solid medium, *phr1*Δ cells give rise to abortive microcolonies containing dead cells.

These results indicate that Chs3p and Chs8p have two different execution points in *phr1*∆ cells, Chs3p acts before Chs8p in protecting the site of germinating *phr1*∆ cells (Figure 10). Notably, these phenotypes also suggest the existence of a strict coordination among Phr1p, Chs3p, Chs8p, and the polarity machinery required at the onset of germ tube emergence and during elongation.

In order to apply these studies to the identification of news drugs, it is important to sort out those transcriptional responses essential for adaptation. Thus, limiting our discussion to the cell wall components identified by several studies as part of the cell wall response, the coordinated increase of chitin, of mannoproteins and of the enzyme that cross-links them to glucan or chitin (Crh11p) might be crucial for viability. However, in *S. cerevisiae* the *gas1*Δ *crh1*Δ mutant *(CRH1* is equivalent to *CRH11* of *C. albicans*) exhibits a phenotype similar to *gas1*Δ suggesting that *CRH1* is not crucial for survival to cell wall stress. No data are available on the phenotype of a combined *phr1*Δ *crh11*Δ mutant of *C. albicans*. On the contrary, the deletion of *GAS1* with *KRE6*, the first gene of the β-(1,6)-glucan synthesis process, is synthetic lethal suggesting that β-(1,6)-glucan is crucial for survival. Chitin and β-(1,6)-glucan synthesis likely compensate for defects in the synthesis/assembly of the core β-(1,3)-glucan mesh.

Among other genes with increased transcription in *phr1*∆ cells, it is worth mentioning: (i) *CPP1*, encoding a dual specificity protein phosphatase homologous to Msg5p of *S. cerevisiae*, which is of interest for its potential role in downregulation of the MAPK pathways, given the promiscuity of action of these protein phosphatases; (ii) *SSK2,* encoding the only MAPKKK of the Hog1p pathway in *C. albicans* and (iii) *FLC2*, in the category of “Transport”, encoding a putative ER membrane transporter that in *S. cerevisiae* is involved in the release of Ca^++^ in response to hyposmotic shock [108].

## 6. Conclusions and Future Outlook

Overall research on the *PHR* multigene family has highlighted the important role of *PHR1* and *PHR2* in *C. albicans* biology and pathogenesis. In a wider vision of human fungal pathogens, GH72 enzymes are essential for virulence and in some instances even for fungus viability. The latter is observed for *GEL4* of *A. fumigatus* [109] and for *GAS1-GAS2* of *C. glabrata* [110], whereas *PHR1* and/or *PHR2* produce a conditional synthetic sick/lethal phenotype [16,61,111].

These findings encourage further studies of the *PHR* family both for increasing basic knowledge of *C. albicans* and also to potentially exploit at Phr1p and Phr2p as molecular targets of new antifungal agents for prophylaxis, therapy of invasive infections, or lock therapy. The armamentarium of antifungal drugs is still limited to three classes: polyenes, azoles, and echinocandin. The advantages of the GH72 enzymes are (i) they have no counterpart in humans; (ii) their extracellular location makes them directly accessible to drugs; (iii) 3D molecular models are available based on the structure of homologous proteins (yeast Gas2p); and (iv) advanced knowledge of the cell wall stress response may provide a means to better monitor drugs’ efficacy or define effective drug target combinations.

The transfer of knowledge from basic biology to therapeutic applications requires an interdisciplinary approach relying on the cooperation of experts in enzymology, glycobiology, structural biology, and bioinformatics. Potential inhibitors would undermine cell wall assembly and in this respect would be the fungal equivalent of penicillin. It is worth mentioning that *PHR* genes/proteins may find applications in medical mycology for species identification by rapid PCR-based tests [112] and for blood tests, since *GAS*/*GEL* proteins are potent fungal antigens that can discriminate patients affected by candidiasis or aspergillosis from healthy individuals and may also have an impact on vaccinations [113,114].

## Figures and Tables

**Table 1:** Catalytic properties of Phr proteins of *Candida albicans* ^1^.

	Phr1p	Phr2p
Activity in vitro	Endo β-(1,3)-glucanase/transglysosylase	Endo β-(1,3)-glucanase/transglysosylase
Temperature optimum	30 °C	30 °C
pH optimum	5.8	3
Half-time of activity decay at 95 °C	10 min	15 min
Minimum length of the donor ^2^	rG9	rG10
Preferred acceptor length	≥L7	≥L7
Residues essential for catalysis ^3^	Glu169 and Glu270	Glu159 and Glu260

^1^ Data refer to GPI-less and His x 6-tagged recombinant forms secreted from *Pichia pastoris* and described in FEMS Yeast Research [36]. ^2^ From [33]. ^3^ From [25] for Phr1p and for Phr2p inferred.
